# Supplementary material for: Effectiveness and Safety of Ustekinumab for Moderate to Severely Active Crohn’s Disease: Results from an Early Access Program in Brazil
Source: J Clin Med. 2022 Oct 31;11(21):6481. doi: 10.3390/jcm11216481 (PMC9654680; doi:10.3390/jcm11216481)
Supplement: Supplementary file 1 [file jcm-11-06481-s001.zip › jcm-1917389-supplementary.pdf]

Supplementary material

Table S1. Initial IV dosing of ustekinumab.

Patient body weight	Recommended dose*	Number of 130 mg usteki-numab vials	Volume of 0.9% w/v so-dium chloride to discard
**≤ 55 kg**	260 mg	2	52 mL
**> 55 kg to ≤ 85 kg**	390 mg	3	78 mL
**> 85 kg**	520 mg	4	104 mL

IV: intravenous. *Approximately 6 mg/kg.

Table S2. Medical history by system organ class and preferred term.

	**Total (*n* = 44)** ***n*** **(%)**
Abnormalities and/or surgical history reported	44 (100.0)
Gastrointestinal disorders	44 (100.0)
Crohn's disease	44 (100.0)
Anal fistula	11 (25.0)
Surgical and medical procedures	27 (61.4)
Ileocolectomy	8 (18.2)
Small bowel resection	8 (18.2)
Musculoskeletal and connective tissue disorders	15 (34.1)
Arthralgia	6 (13.6)
Blood and lymphatic system disorders	11 (25.0)
Anemia	8 (18.2)
Psychiatric disorders	9 (20.5)
Anxiety	5 (11.4)
Infections and infestations	8 (18.2)
Skin and subcutaneous tissue disorders	8 (18.2)
Vascular disorders	8 (18.2)
Hypertension	8 (18.2)
Metabolism and nutrition disorders	7 (15.9)
Cardiac disorders	5 (11.4)
Reproductive system and breast disorders	5 (11.4)
Social circumstances	5 (11.4)
Immune system disorders	4 (9.1)
Renal and urinary disorders	4 (9.1)
Respiratory, thoracic, and mediastinal disorders	4 (9.1)
Hepatobiliary disorders	3 (6.8)
Injury, poisoning, and procedural complications	3 (6.8)
Nervous system disorders	3 (6.8)
Endocrine disorders	2 (4.5)
Investigations	2 (4.5)
Congenital, familial, and genetic disorders	1 (2.3)
General disorders and administration site conditions	1 (2.3)
Pregnancy, puerperium, and perinatal conditions	1 (2.3)

Table S3. Chemistry results.

	**At Baseline (*n* = 44)**	**Week 8 (*n* = 44)**	**Week 16/20 (*n* = 42)**	**Week 40/44 (*n* = 41)**	**At Week 80 (*n* = 34)**	**Safety Fol-low-up (*n* = 34)**
Sample collected for analysis, *n* (%)						
Yes	44 (100.0)	39 (88.6)	42 (100.0)	40 (97.6)	33 (97.1)	28 (84.8)
No	0	5	0	1	1	5
Total	44	44	42	41	34	33
Missing values	0	0	0	0	0	1
ALT, *n* (%)	44 (100.0)	39 (100.00)	42 (100.0)	40 (100.0)	33 (100.00)	28 (100.0)
Mean (SD) (U/L)	18.85 (10.86)	21.62 (13.27)	22.45 (14.09)	23.98 (21.33)	26.1 (24.87)	41.03 (77.91)
Median (Q1-Q3) (U/L)	15 (11.7-14.86)	16 (12-20)	19.5 (12-16.32)	16.81 (11.5-21.5)	17.28 (13-27)	19.5 (14.5-25.45)
Normal	39 (88.6)	30 (76.9)	34 (81.0)	32 (80.0)	26 (78.8)	23 (82.1)

	At Baseline (*n* = 44)	Week 8 (*n* = 44)	Week 16/20 (*n* = 42)	Week 40/44 (*n* = 41)	At Week 80 (*n* = 34)	Safety Follow-up (*n* = 34)
Abnormal (clinically significant)	0 (0.0)	0 (0.0)	0 (0.0)	0 (0.0)	1 (3.0)	2 (7.1)
AST, *n* (%)	44 (100.0)	39 (100.0)	42 (100.0)	40 (100.00)	33 (100.0)	28 (100.0)
Mean (SD) (U/L)	19.4 (5.86)	21.2 (10.16)	21.33 (7.22)	22.02 (9.05)	24.77 (12.45)	30.11 (30.23)
Median (Q1-Q3) (U/L)	18.24 (15.5-24)	18.9 (15-27.95)	20.35 (16-27)	20 (15.5-26.5)	21 (18-28)	21 (16.72-26.08)
Normal	44 (100.0)	36 (92.3)	41 (97.6)	38 (95.0)	30 (90.9)	24 (85.7)
Abnormal (clinically significant)	0 (0.0)	0 (0.0)	0 (0.0)	0 (0.0)	0 (0.0)	1 (3.6)
Alkaline phosphatase, *n* (%)	38 (86.4)	37 (94.9)	41 (97.6)	40 (100.0)	31 (93.9)	27 (96.4)
Mean (SD) (U/L)	112.47 (64.54)	111.52 (70.35)	111.55 (66.84)	110.49 (73.58)	120.48 (88.89)	152.1 (185.59)
Median (Q1-Q3) (U/L)	94.25 (55-153)	98.7 (58-134)	98 (61-129)	82 (57-147.44)	83 (55-143.33)	82 (58-176.28)
Normal	32 (84.2)	32 (86.5)	34 (82.9)	35 (87.5)	25 (80.6)	23 (85.2)
Abnormal (clinically significant)	0 (0.0)	0 (0.0)	0 (0.0)	1 (2.5)	0 (0.0)	1 (3.7)
Total bilirubin, *n* (%)	44 (100.0)	37 (94.9)	41 (97.6)	40 (100.0)	33 (100.0)	28 (100.0)
Mean (SD) (mg/dL)	0.53 (0.32)	0.51 (0.37)	0.55 (0.33)	0.55 (0.36)	0.61 (0.55)	0.8 (1.35)
Median (Q1-Q3) (mg/dL)	0.5 (0.32-0.6)	0.4 (0.3-0.54)	0.4 (0.36-0.7)	0.5 (0.34-0.6)	0.5 (0.3-0.67)	0.44 (0.3-0.7)
Normal	42 (95.5)	35 (94.6)	39 (95.1)	38 (95.0)	30 (90.9)	21 (75.0)
Abnormal (clinically significant)	0 (0.0)	0 (0.0)	0 (0.0)	0 (0.0)	0 (0.0)	1 (3.6)
Direct bilirubin, *n* (%)	44 (100.0)	37 (94.9)	41 (97.6)	40 (100.0)	33 (100.0)	28 (100.0)
Mean (SD) (mg/dL)	0.22 (0.17)	0.23 (0.17)	0.25 (0.19)	0.21 (0.14)	0.24 (0.17)	0.42 (1.11)
Median (Q1-Q3) (mg/dL)	0.2 (0.1-0.3)	0.2 (0.11-0.3)	0.2 (0.1-0.3)	0.2 (0.1-0.3)	0.2 (0.1-0.3)	0.15 (0.1-0.3)
Normal	39 (88.6)	34 (91.9)	34 (82.9)	38 (95.0)	30 (90.9)	25 (89.3)
Abnormal (clinically significant)	0 (0.0)	0 (0.0)	0 (0.0)	0 (0.0)	0 (0.0)	1 (3.6)
Albumin, *n* (%)	39 (88.6)	37 (94.9)	41 (97.6)	39 (97.5)	30 (90.9)	24 (85.7)
Mean (SD) (g/dL)	3.92 (0.48)	3.96 (0.43)	3.9 (0.45)	3.9 (0.44)	4.14 (0.43)	4.19 (0.47)
Median (Q1-Q3) (g/dL)	3.87 (3.7-4.21)	3.98 (3.8-4.2)	3.93 (3.7-4.2)	4 (3.8-4.2)	4.3 (3.9-4.4)	4.3 (4.01-4.49)
Normal	36 (92.3)	34 (91.9)	37 (90.2)	33 (84.6)	27 (90.0)	22 (91.7)
Abnormal (clinically significant)	1 (2.6)	1 (2.7)	1 (2.4)	1 (2.6)	1 (3.3)	1 (4.2)
Total protein, *n* (%)	39 (88.6)	37 (94.9)	37 (88.1)	39 (97.5)	33 (100.0)	24 (85.7)
Mean (SD) (g/dL)	7.07 (1.05)	7.17 (0.9)	7.12 (0.88)	6.99 (0.88)	7.04 (0.86)	7.05 (0.96)
Median (Q1-Q3) (g/dL)	7 (6.4-7.8)	7.2 (6.88-7.8)	7.1 (6.7-7.5)	6.9 (6.6-7.7)	7.1 (6.7-7.6)	7.1 (6.7-7.72)
Normal	26 (66.7)	31 (83.8)	30 (81.1)	32 (82.1)	27 (81.8)	17 (70.8)
Abnormal (clinically significant)	0 (0.0)	1 (2.7)	1 (2.7)	1 (2.6)	1 (3.0)	1 (4.2)
Chloride, *n* (%)	31 (70.5)	32 (82.1)	37 (88.1)	34 (85.0)	33 (100.0)	27 (96.4)
Mean (SD) (mEq/L)	100.66 (4.33)	102.09 (3.68)	102.19 (3.73)	102.09 (3.53)	102.78 (2.69)	102.12 (2.67)
Median (Q1-Q3) (mEq/L)	101 (98-104.15)	102.36 (100-104.97)	102 (100.2-104.98)	102 (100-104.05)	102.66 (101-105)	102.37 (100-104)
Normal	25 (80.6)	30 (93.8)	33 (89.2)	30 (88.2)	31 (93.9)	26 (96.3)
Abnormal (clinically significant)	0 (0.0)	0 (0.0)	0 (0.0)	0 (0.0)	0 (0.0)	0 (0.0)
Urea, *n* (%)	39 (88.6)	37 (94.9)	41 (97.6)	40 (100.0)	33 (100.0)	28 (100.0)
Mean (SD) (mg/dL)	27.87 (9.82)	26.41 (9.13)	27.86 (8.73)	25.27 (10.17)	27.87 (7.63)	29.12 (7.04)
Median (Q1-Q3) (mg/dL)	26 (22-33.49)	24.29 (20-31)	26 (22.8-35)	24.5 (19.26-30.55)	25.8 (23-31)	29 (23.85-34.5)
Normal	35 (89.7)	35 (94.6)	38 (92.7)	38 (95.0)	31 (93.9)	26 (92.9)
Abnormal (clinically significant)	0 (0.0)	0 (0.0)	0 (0.0)	0 (0.0)	0 (0.0)	0 (0.0)
Creatinine, *n* (%)	44 (100.0)	37 (94.9)	41 (97.6)	40 (100.0)	32 (97.0)	28 (100.0)
Mean (SD) (mg/dL)	0.81 (0.21)	0.83 (0.22)	0.87 (0.22)	0.85 (0.25)	0.9 (0.2)	0.89 (0.22)

	At Baseline (*n* = 44)	Week 8 (*n* = 44)	Week 16/20 (*n* = 42)	Week 40/44 (*n* = 41)	At Week 80 (*n* = 34)	Safety Follow-up (*n* = 34)
Median (Q1-Q3) (mg/dL)	0.8 (0.67-0.97)	0.8 (0.7-0.99)	0.9 (0.7-1)	0.8 (0.7-1)	0.9 (0.8-1)	0.9 (0.7-1.08)
Normal	39 (88.6)	32 (86.5)	37 (90.2)	35 (87.5)	30 (93.8)	25 (89.3)
Abnormal (clinically significant)	0 (0.0)	0 (0.0)	0 (0.0)	0 (0.0)	0 (0.0)	0 (0.0)
Sodium performed, *n* (%)	37 (84.1)	37 (94.9)	40 (95.2)	40 (100.0)	32 (97.0)	28 (100.0)
Mean (SD) (mmol/L)	140.19 (3.25)	140.47 (2.15)	140.12 (2.52)	139.74 (2.87)	141.07 (2.31)	140.59 (2.32)
Median (Q1-Q3) (mmol/L)	140 (138-143)	141 (140-142)	140 (139-141.62)	140.16 (138-141.65)	141 (139-142.51)	140 (139-142)
Normal	34 (91.9)	37 (100.0)	39 (97.5)	39 (97.5)	31 (96.9)	26 (92.9)
Abnormal (clinically significant)	0 (0.0)	0 (0.0)	0 (0.0)	0 (0.0)	0 (0.0)	0 (0.0)
Potassium, *n* (%)	36 (81.8)	37 (94.9)	41 (97.6)	40 (100.0)	32 (97.0)	28 (100.0)
Mean (SD) (mmol/L)	4.1 (0.49)	4.19 (0.34)	4.3 (0.41)	4.24 (0.42)	4.22 (0.49)	4.23 (0.49)
Median (Q1-Q3) (mmol/L)	4.09 (3.67-4.5)	4.19 (3.9-4.46)	4.3 (4-4.6)	4.2 (4-4.5)	4.16 (3.81-4.46)	4.23 (3.9-4.5)
Normal	34 (94.4)	37 (100.0)	41 (100.0)	38 (95.0)	30 (93.8)	26 (92.9)
Abnormal (clinically significant)	0 (0.0)	0 (0.0)	0 (0.0)	0 (0.0)	0 (0.0)	0 (0.0)
Calcium, *n* (%)	32 (72.7)	35 (89.7)	41 (97.6)	36 (90.0)	33 (100.0)	27 (96.4)
Mean (SD) (mg/dL)	6.38 (2.19)	6.05 (2.3)	6.88 (2.32)	6.5 (2.36)	6.17 (2.38)	5.95 (2.26)
Median (Q1-Q3) (mg/dL)	4.9 (4.42-8.72)	4.92 (4.48-9)	7.5 (4.64-9.14)	4.92 (4.44-8.9)	4.68 (4.36-8.7)	4.76 (4.2-9)
Normal	19 (59.4)	27 (77.1)	33 (80.5)	25 (69.4)	18 (54.5)	19 (70.4)
Abnormal (clinically significant)	0 (0.0)	0 (0.0)	0 (0.0)	0 (0.0)	0 (0.0)	0 (0.0)
Phosphorus, *n* (%)	30 (68.2)	34 (87.2)	41 (97.6)	36 (90.0)	33 (100.0)	28 (100.0)
Mean (SD) (mg/dL)	3.54 (0.59)	3.68 (0.65)	3.6 (0.64)	3.49 (0.71)	3.65 (0.74)	3.47 (0.49)
Median (Q1-Q3) (mg/dL)	3.6 (3.3-3.9)	3.5 (3.11-4.2)	3.7 (3.1-3.95)	3.52 (3.03-3.95)	3.54 (3-4.34)	3.5 (3.1-3.8)
Normal	27 (90.0)	31 (91.2)	38 (92.7)	33 (91.7)	30 (90.9)	27 (96.4)
Abnormal (clinically significant)	0 (0.0)	0 (0.0)	0 (0.0)	0 (0.0)	0 (0.0)	0 (0.0)
